# Supplementary material for: Multivalent in vivo delivery of DNA-encoded bispecific T cell engagers effectively controls heterogeneous GBM tumors and mitigates immune escape
Source: Mol Ther Oncolytics. 2023 Feb 16;28:249–63. doi: 10.1016/j.omto.2023.02.004 (PMC10006507; doi:10.1016/j.omto.2023.02.004)
Supplement: Document S1. Figures S1 and S2 [file mmc1.pdf]

OMTO, Volume *28*

Supplemental information

Multivalent *in vivo* delivery of DNA-encoded bispecific T cell engagers effectively controls heterogeneous GBM tumors and mitigates immune escape

Daniel H. Park, Kevin Liaw, Pratik Bhojnagarwala, Xizhou Zhu, Jihae Choi, Ali R. Ali, Devivasha Bordoloi, Ebony N. Gary, Ryan P. O'Connell, Abhijeet Kulkarni, Diana Guimet, Trevor Smith, Alfredo Perales-Puchalt, Ami Patel, and David B. Weiner

Figure S1

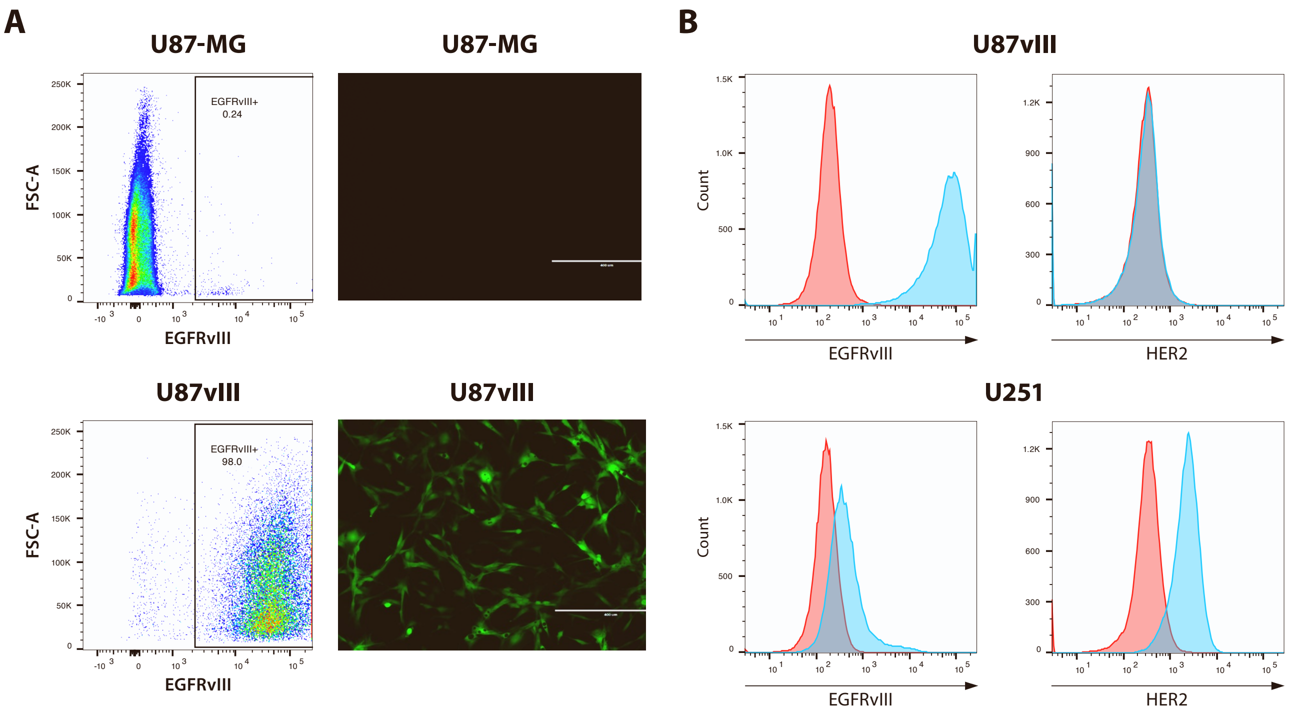

Figure S1. GBM cell lines and antigen expression

(A) Flow cytometry data and fluorescent images showing EGFRvIII expression in wildtype U87 cells and U87vIII cells. (B) Flow cytometry data showing EGFRvIII and HER2 expression in U87vIII cells and U251 cells.

Figure S2

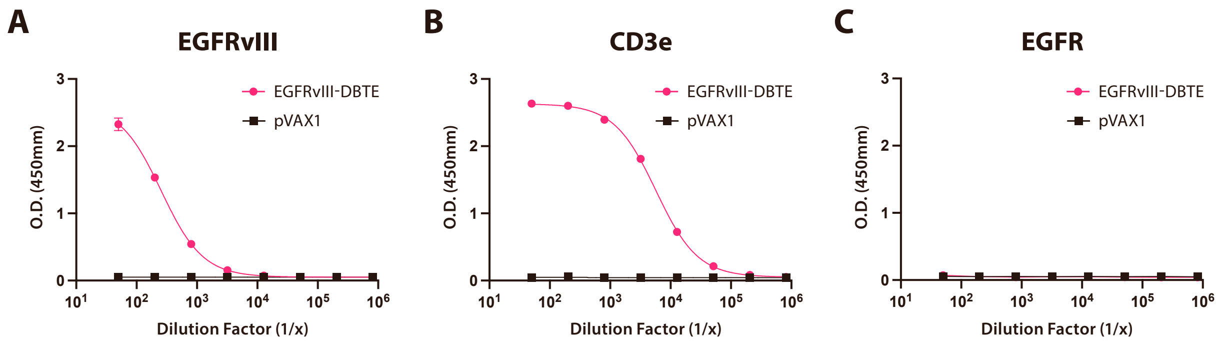

Figure S2. Binding ELISA of EGFRvIII-DBTE

Binding ELISA of EGFRvIII-DBTE (transfection supernatant) against recombinant (A) EGFRvIII, (B) CD3e, and (C) wildtype EGFR.

Video S1

Video S1. Time-lapse video of T cell-mediated cytotoxicity of EGFRvIII-DBTE

Time-lapse video of T cell mediated cytotoxicity against U87vIII cells upon addition of day-14 serum of NSG mouse treated with pVAX1 or EGFRvIII-DBTE. U87vIII is shown in green (GFP). Caspase-3 induction is shown in blue. CD69 activation is shown in red. Hour marks at the top left corner indicate incubation time after addition of T cells and mouse sera.
