# Supplementary material for: Multivalent in vivo delivery of DNA-encoded bispecific T cell engagers effectively controls heterogeneous GBM tumors and mitigates immune escape
Source: Mol Ther Oncolytics. 2023 Feb 16;28:249–63. doi: 10.1016/j.omto.2023.02.004 (PMC10006507; doi:10.1016/j.omto.2023.02.004)
Supplement: Document S2. Article plus supplemental information [file mmc3.pdf]

Molecular Therapy
Oncolytics
Original Article

Multivalent *in vivo* delivery of DNA-encoded bispecific T cell engagers effectively controls heterogeneous GBM tumors and mitigates immune escape

Daniel H. Park,[1,2] Kevin Liaw,[2] Pratik Bhojnagarwala,[2] Xizhou Zhu,[2] Jihae Choi,[2] Ali R. Ali,[2] Devivasha Bordoloi,[2] Ebony N. Gary,[2] Ryan P. O'Connell,[1,2] Abhijeet Kulkarni,[2] Diana Guimet,[3] Trevor Smith,[3] Alfredo Perales-Puchalt,[2] Ami Patel,[2] and David B. Weiner[1,2]

[1]Perelman School of Medicine, The University of Pennsylvania, Philadelphia, PA, USA; [2]Vaccine and Immunotherapy Center, The Wistar Institute, Philadelphia, PA, USA; [3]Inovio Pharmaceuticals, Plymouth Meeting, PA 19462, USA

Glioblastoma multiforme (GBM) is among the most difficult cancers to treat with a 5-year survival rate less than 5%. An immunotherapeutic vaccine approach targeting GBM-specific antigen, EGFRvIII, previously demonstrated important clinical impact. However, immune escape variants were reported in the trial, suggesting that multivalent approaches targeting GBM-associated antigens may be of importance. Here we focused on multivalent *in vivo* delivery of synthetic DNA-encoded bispecific T cell engagers (DBTEs) targeting two GBM-associated antigens, EGFRvIII and HER2. We designed and optimized an EGFRvIII-DBTE that induced T cell-mediated cytotoxicity against EGFRvIII-expressing tumor cells. *In vivo* delivery in a single administration of EGFRvIII-DBTE resulted in durable expression over several months in NSG mice and potent tumor control and clearance in both peripheral and orthotopic animal models of GBM. Next, we combined delivery of EGFRvIII-DBTEs with an HER2-targeting DBTE to treat heterogeneous GBM tumors. *In vivo* delivery of dual DBTEs targeting these two GBM-associated antigens exhibited enhanced tumor control and clearance in a heterogeneous orthotopic GBM challenge, while treatment with single-target DBTE ultimately allowed for tumor escape. These studies support that combined delivery of DBTEs, targeting both EGFRvIII and HER2, can potentially improve outcomes of GBM immunotherapy, and such multivalent approaches deserve additional study.

INTRODUCTION

Glioblastoma multiforme (GBM) is the most lethal and aggressive glioma in adults with a 5-year survival rate of less than 5%.[1] With a standard of care that comprises surgical resection, radiation, and chemotherapy, the median survival remains 15 months for GBM patients. Approximately 40% of the patients have unresectable GBM and show poorer prognosis due to high recurrence rate.[2] Currently, there is no Food and Drug Administration (FDA)-approved immunotherapy for GBM patients. The poor prognosis and the lack of alternative therapy illustrate the highly unmet clinical need of new therapies for GBM patients.

Recently, immunotherapies targeting epidermal growth factor receptor (EGFR) variant III (EGFRvIII) are receiving attention as potential treatment options for GBM. EGFRvIII is the most frequent mutant form of EGFR, which results from in-frame deletion of the EGF ligand-binding domain.[3] EGFRvIII is an oncogenic, tumor-specific surface antigen that is present on up to 30% of newly diagnosed GBM cases and is undetectable in normal tissues, making it an ideal target for immunotherapy.[3,4] EGFRvIII-targeted approaches previously tested in clinical trials include chimeric antigen receptor T cells (CAR-T) as well as studies with a peptide vaccine strategy.[5,6] However, they so far have demonstrated limited clinical benefits beyond the standard of care, with one obstacle reported of targeted antigen loss, resulting in tumor escape in treated patients.

Immune escape poses a significant challenge for antigen-targeted immunotherapies for GBM, which manifests a heterogeneous antigen landscape. GBM exhibits various degrees of antigenic heterogeneity. Clinical studies reveal that the expressions of antigens such as EGFRvIII and HER2 are highly heterogeneous in GBM patient samples.[7,8] The antigen heterogeneity could be driven in part from tumor cells that evade immune surveillance by downregulation, mutation, deletion of antigen, and selective survival of antigen-negative tumor subpopulations.[9–11] Such mechanisms of antigen escape create challenges for single antigen-targeted approaches in effectively eliminating the entire tumor burden and preventing recurrence. Thus,

Received 4 January 2023; accepted 10 February 2023;
https://doi.org/10.1016/j.omto.2023.02.004.

Correspondence: David B. Weiner, Vaccine and Immunotherapy Center, The Wistar Institute, Philadelphia, PA, USA.
E-mail: dweiner@wistar.org

This is an open access article under the CC BY license (http://creativecommons.org/licenses/by/4.0/).

strategies that can target multiple tumor antigens simultaneously may be of importance for GBM patients.

Bispecific T cell engagers (BTEs), which target antigen-specific T cell-mediated anti-tumor cytotoxicity, are being studied for targeting solid tumors in preclinical and clinical studies.[12,13] An EGFRvIII-targeting BTE was studied in an animal model of GBM, which demonstrated moderate tumor control as well as survival through delivery of 16 consecutive daily doses.[14] Improving potency and *in vivo* pharmacokinetics are important for further development. Direct *in vivo* delivery of BTEs with more durable expression remains an important goal for study in therapeutic models of GBM. Such an approach could simplify clinical translation, providing patient benefit by improved pharmacokinetics likely with lower costs.

In a preliminary study, we described a DNA-encoded BTE (DBTE) targeting ovarian cancer in a peripheral challenge model.[15] Here we build on this work focusing on engineering a new *in vivo*-produced EGFRvIII-targeting DBTE (EGFRvIII-DBTE) first as a monotherapy for direct *in vivo* treatment for GBM in both peripheral and orthotopic challenge animal models. We show the *in vivo* expression of the EGFRvIII-DBTE, specificity, T cell-mediated cytotoxicity, and efficacy in challenge models of GBM. We report that a single injection of EGFRvIII-DBTE exhibited durable *in vivo* expression and potent tumor regression and clearance in mice.

We advance the study to describe a delivery of multiple DBTEs as a potential combination therapy for heterogeneous GBM. The GBM-associated antigens, EGFRvIII and HER2, are expressed in up to 30% and 80% of GBM cases, respectively.[3,16] We hypothesize that a combination approach of the EGFRvIII-DBTE in conjunction with HER2-targeting DBTE (HER2-DBTE) would limit GBM immune escape *in vivo*. We developed an EGFRvIII$^+$/HER2$^+$ heterogeneous GBM model to demonstrate that a co-administration of EGFRvIII-DBTE and HER2-DBTE dramatically enhanced tumor suppression in the heterogeneous GBM challenge as compared with single DBTEs. These findings suggest targeting multiple antigens, as was studied here, likely provide more potent tumor targeting and limit immune escape in GBM as well as other diverse cancers supporting improved patient benefit.

RESULTS

Design and *in vitro* expression of EGFRvIII-targeted DNA-encoded bispecific T cell engager

To develop an EGFRvIII-targeted DNA-encoded bispecific T cell engager (EGFRvIII-DBTE), we identified variable fragment sequences for an EGFRvIII-binding antibody[17] and a humanized CD3-binding antibody (clone UCHT-1). The sequences were modified to generate scFv sequences through codon optimization specific for *in vivo* expression and fusion with a GS linker (Figure 1A). For improved expression in mammalian cells, we added a human immunoglobulin (Ig)E leader sequence to the N terminus, as we have previously described,[15] and encoded this construct in a modified pVAX1 expression vector. This EGFRvIII-DBTE was expressed *in vitro* using an Expi293 expression system. The supernatant from transfection studies was examined by western blotting to initially confirm expression, using pVAX1 empty vector as a negative control (Figure 1B).

Generation of EGFRvIII-expressing tumor cells

To develop a therapeutic model of GBM, we generated a GBM cell line stably expressing EGFRvIII. We transfected Phoenix-AMPHO cells with a DNA plasmid encoding extracellular sequence of EGFRvIII, which produced gamma-retrovirus containing the EGFRvIII construct and then used the virus-containing media to transduce U87-MG, an aggressive malignant glioma cell line to generate a GBM cell line stably expressing EGFRvIII. The construct included a GFP reporter, allowing for identification of tumor cells *in vitro* as well as *in vivo*. The EGFRvIII-expressing U87-MG cells (U87vIII) were sorted via GFP and subcloned allowing for generation of a homogeneous stable positive population that then was validated by flow cytometry (Figure S1A).

EGFRvIII-DBTE binds EGFRvIII and CD3

To examine the binding properties of EGFRvIII-DBTE, we incubated U87vIII cells with EGFRvIII-DBTE and stained them with an anti-human IgG F(ab')2 fragment secondary antibody. For CD3-binding, primary human T cells were used. Supernatants collected from empty vector pVAX1 and HER2-targeted DBTE (HER2-DBTE) were used as controls. By flow cytometry analysis, we observed that EGFRvIII-DBTE engaged with both U87vIII and human T cells (Figure 1C). Binding specificity was also confirmed by ELISA in which EGFRvIII-DBTE did not bind to wild-type EGFR (Figure S2). We further examined EGFRvIII-DBTE's ability to form an immunological synapse between the target and effector cells by co-incubating U87vIII cells and T cells in the presence of EGFRvIII-DBTE and examined cultures for T cells engaging with U87vIII cells. We gated on the tumor population and observed a double-positive population of GFP (U87vIII) and CD3 (T cells) in the presence of EGFRvIII-DBTE, indicating that T cells were engaging tumor cells (Figure 1D). This engagement was not observed in the presence of an irrelevant control DBTE. In another assay, we plated U87vIII cells in a tissue culture plate with T cells and observed by fluorescent microscopy that EGFRvIII-DBTE induced T cells to cluster around the target cells (Figure 1E). These data support that *in vitro*-expressed EGFRvIII-DBTE binds to both EGFRvIII and CD3, facilitating T cells to bind to target cells with high specificity.

EGFRvIII-DBTE cytotoxicity against EGFRvIII-expressing tumor cells

We examined if EGFRvIII-DBTE can induce T cell-mediated cytotoxicity against U87vIII cells. We used the xCelligence RTCA system, which uses gold biosensors at the bottom of the special plate to continuously and non-invasively measure the relative cell counts by impedance differential created by cell attachment to the plate. U87vIII cells were plated in a 96-well E-plate and primary human T cells were added at an E:T ratio of 10:1 and EGFRvIII-DBTE at 10 ng/mL. The viability of U87vIII cells was measured in real time for 48 h by xCelligence RTCA analyzer. As a result, EGFRvIII-DBTE

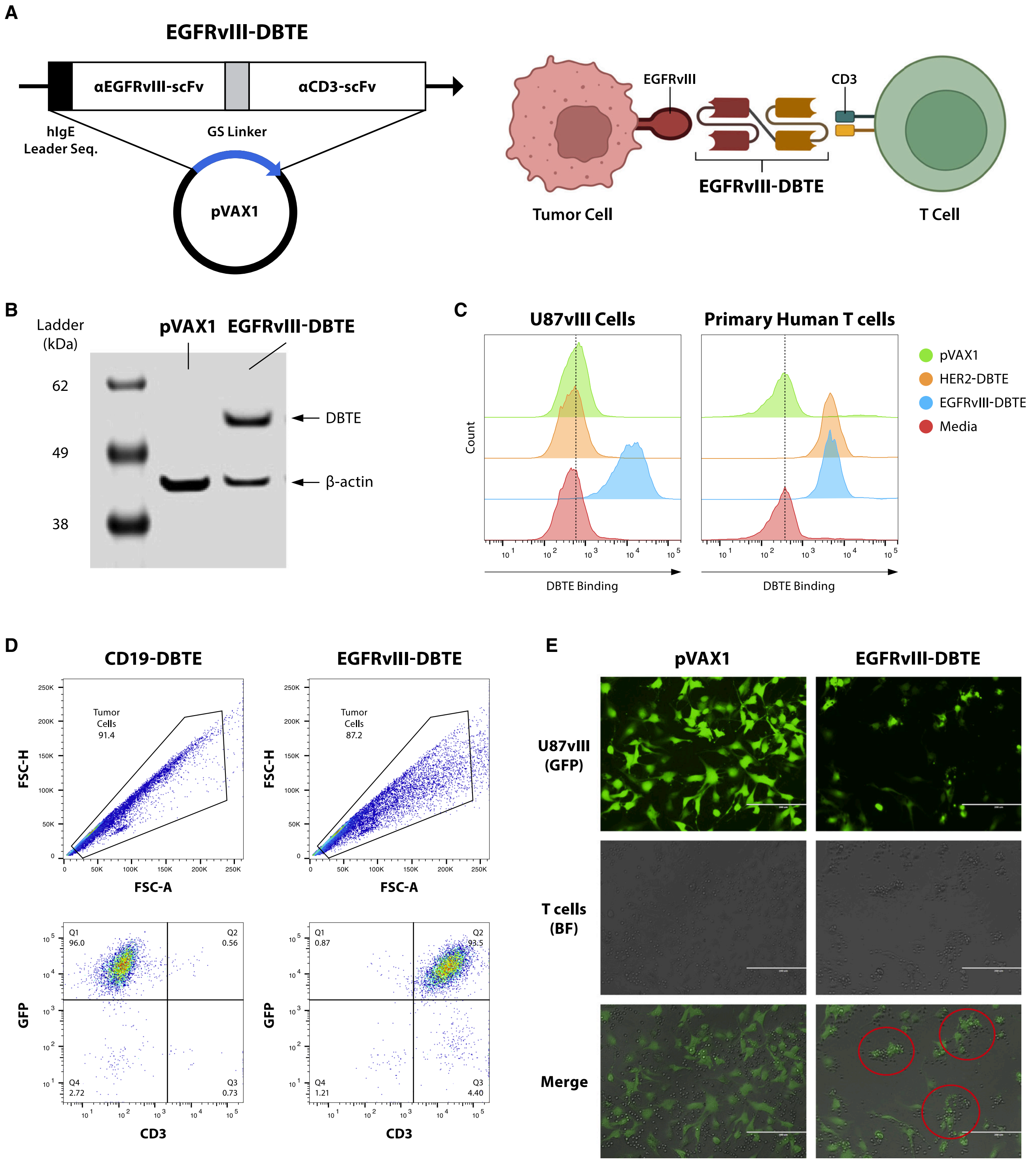

Figure 1. Design, *in vitro* expression, and binding assessment of EGFRvIII-DBTE

(A) Design and structure of EGFRvIII-DBTE. (B) Western blot using supernatant of Expi293F cells transfected with EGFRvIII-DBTE or pVAX1 (vehicle control). (C) Flow cytometry data showing on-cell binding activities of EGFRvIII-DBTE to U87vIII cells and human T cells. HER2-DBTE was used as an isotype control. (D) Flow cytometry data showing T cell-bound U87vIII cells in the presence of EGFRvIII-DBTE. GFP is an EGFRvIII reporter. CD19-DBTE was used as an isotype control. (E) Microscopic images showing T cell clustering around the U87vIII cells in a tumor-killing assay (5-h incubation).

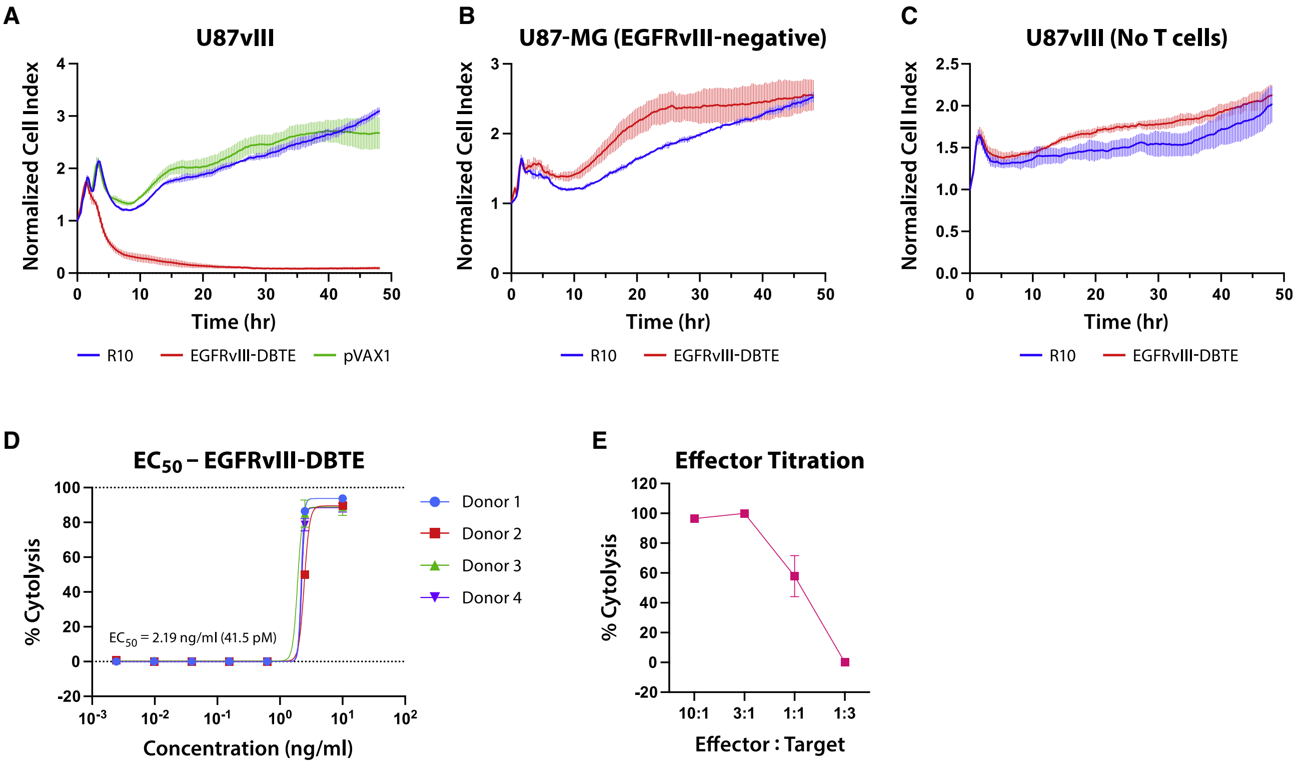

Figure 2. T cell-mediated cytotoxicity of EGFRvIII-DBTE

Real-time target cell viability in tumor-killing assay upon addition of EGFRvIII-DBTE (A) with human T cells, (B) without human T cells, and (C) with human T cells in the absence of EGFRvIII on target cells (U87-MG). (D) Percent cytolysis data in 48-h tumor-killing assay using a series of concentrations of EGFRvIII-DBTE. T cells from four different donors were used to determine EC_{50} value. E:T ratio was 10:1 for (A)–(D). (E) Percent cytolysis data in 48-h tumor-killing assay using a series of E:T ratios.

induced potent T cell-mediated cytotoxicity against U87vIII cells (Figure 2A). We observed that EGFRvIII-DBTE did not induce cytotoxicity without T cells (Figure 2B) or against EGFRvIII-negative U87 cells (Figure 2C). To assess a half maximal effective concentration (EC_{50}) value of EGFRvIII-DBTE, we examined % cytolysis using EGFRvIII-DBTE at a series of concentrations in 48-h tumor-killing assay. Using primary T cells from four different donors, we determined that EC_{50} of EGFRvIII-DBTE against U87vIII cells was potent at 2.19 ng/mL, which is equivalent to 41.5 pM (Figure 2D). In addition, we examined the potency of EGFRvIII-DBTE in lower E:T ratios and observed that EGFRvIII-DBTE induced significant cytotoxicity at the low E:T ratio of 1:1 (Figure 2E).

Targeting tumors by EGFRvIII-DBTE drives T cell activation

To explore EGFRvIII-DBTE's ability to enhance T cell functions, we examined activation markers and cytokine release in primary T cells after stimulation by EGFRvIII-DBTE. In the tumor-killing assay described above, we added fluorochrome-conjugated CD69 antibody and caspase-3 dye and monitored T cell activity. Upon addition of NOD scid gamma (NSG) mouse sera, which was treated with an intramuscular (IM) injection of 100 μg of EGFRvIII-DBTE followed by EP, we observed CD69 activation in T cells that was focused on target-bound T cells, as well as activation of the caspase-3 pathway in target cells (Figure 3A). The cell counts of GFP^+ target cells decreased significantly by 12 h when EGFRvIII-DBTE was added (Figure 3B). CD69 activation (Figure 3C) and caspase-3 induction (Figure 3D) was rapid with initiation within 6 h and persistent throughout 48-h incubation, showing increasing signals for CD69. Video S1 is a movie showing side-by-side comparison between pVAX1 and EGFRvIII-DBTE in the tumor-killing assay. Here, we observed that T cells migrated toward the target tumor cells during CD69 activation (Video S1).

Next, cytokine responses were examined in T cells using the tumor-killing assay. T cells were collected after a 24-h incubation with U87vIII cells in the presence of EGFRvIII-DBTE and then analyzed by flow cytometry. CD19-DBTE was used as an isotype control. We observed that $CD4^+$ and $CD8^+$ T cell populations both exhibited increased secretion of interferon (IFN)-γ, tumor necrosis factor (TNF)-α, and interleukin (IL)-2, which are associated with anti-tumor activities (Figure 3E). $CD8^+$ T cells displayed upregulation of IFN-γ and TNF-α secretion while $CD4^+$ T cells exhibited an upregulation of IL-2 and TNF-α secretion. Both $CD4^+$ and $CD8^+$ T cells showed activation of CD107a, a marker for degranulation, with a greater response observed in $CD8^+$ T cells (Figure 3F).

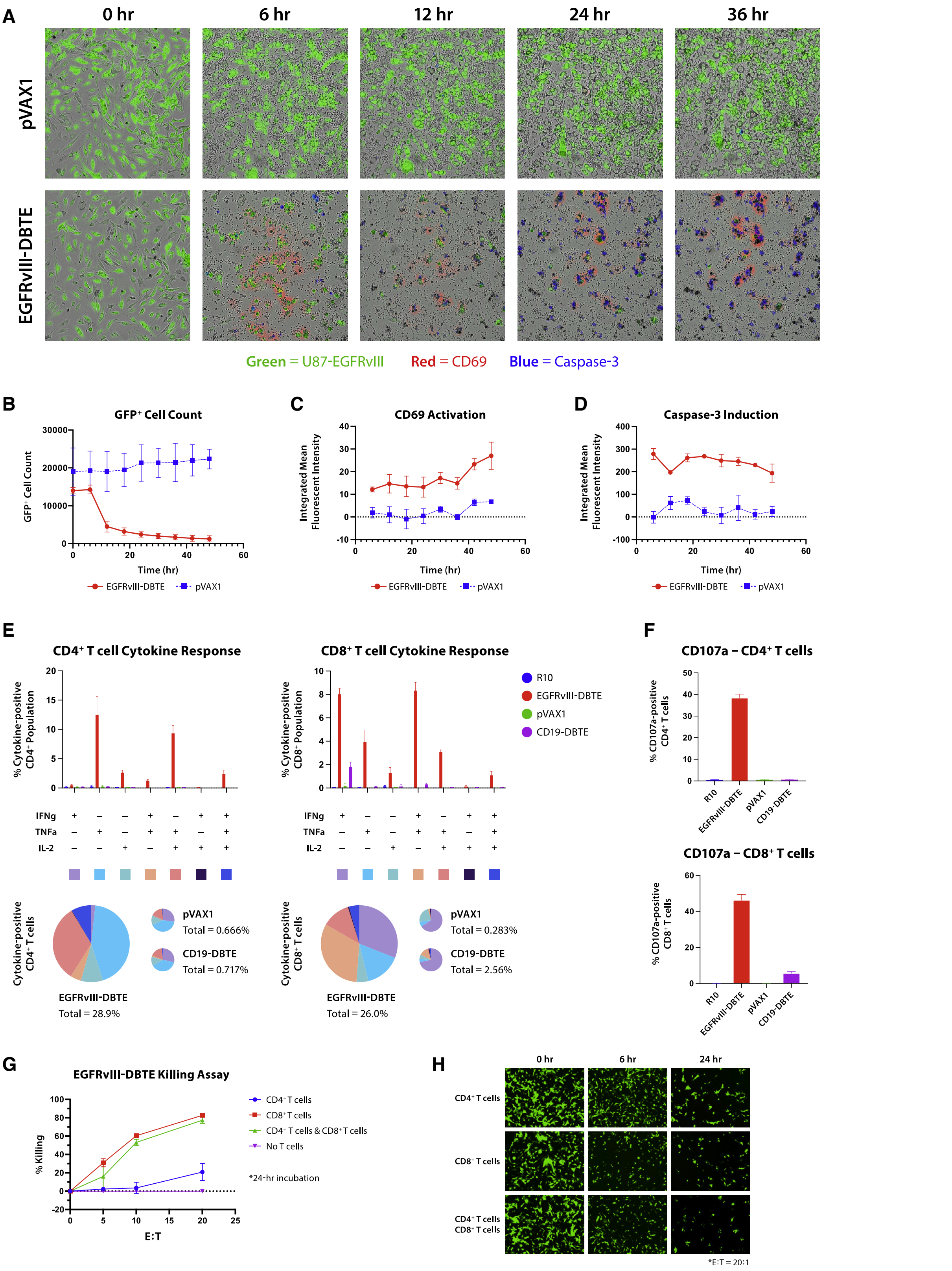

(legend on next page)

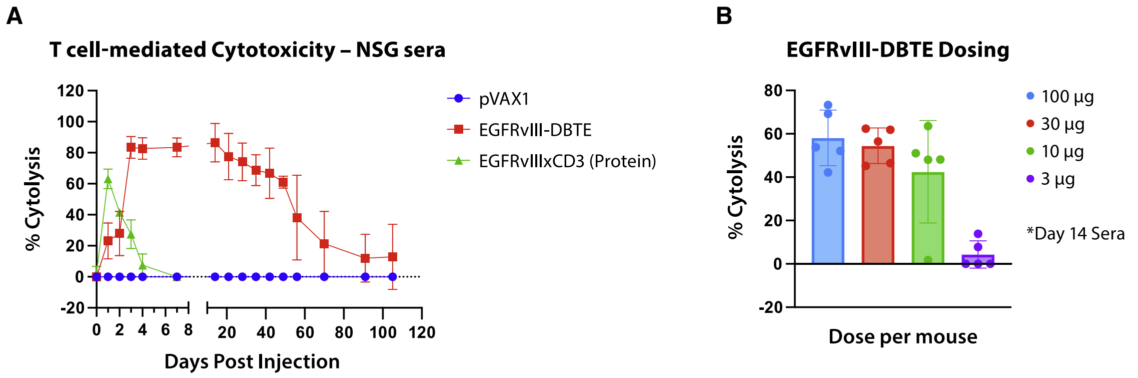

Figure 4. *In vivo* expression of EGFRvIII-DBTE

(A) T cell-mediated cytotoxicity assay against U87vIII cells using NSG mouse sera treated with a single injection (100 μg) of pVAX1, EGFRvIII-DBTE, or recombinant EGFRvIIIxCD3 antibody. (B) T cell-mediated cytotoxicity assay against U87vIII cells using NSG mouse sera treated with various doses of EGFRvIII-DBTE. % Cytolysis data after 48-h incubation were plotted for both (A) and (B).

$CD4^+$ and $CD8^+$ T cells were sorted and used as effector cells in a tumor-killing assay to examine their independent cytotoxicity. We observed robust cytotoxicity from $CD8^+$ T cells (Figure 3G). Importantly, $CD4^+$ T cells also showed significant but lower cytotoxicity at a high E:T ratio of 20:1 (Figure 3G). By fluorescent microscopy, we observed that the onset of tumor cytolysis was more rapid in $CD8^+$ T cells than in $CD4^+$ T cells (Figure 3H). These data support that EGFRvIII-DBTE drives anti-tumor activation of both $CD8^+$ and $CD4^+$ T cells that can contribute to bispecific killing potential against tumor targets with slower kinetics.

In vivo expression of EGFRvIII-DBTE

To determine functionality of EGFRvIII-DBTE expressed *in vivo*, we injected a single dose of EGFRvIII-DBTE or pVAX1 in the tibialis anterior (TA) muscle of NSG mice using electroporation (EP), as previously described.[18] Sera were collected over a period of 105 days and EGFRvIII-DBTE activity was studied in 48-h T cell-mediated cytotoxicity assays against U87vIII cells to monitor tumor killing over time. We observed that a single injection of 100 μg of EGFRvIII-DBTE produced a durable expression in NSG mice of more than 100 days (Figure 4A). For comparison, sera of NSG mice given an intraperitoneal (i.p.) injection of 100 μg of protein EGFRvIIIxCD3 BTE was included as a control. Cytotoxicity of the i.p.-delivered protein EGFRvIIIxCD3 BTE peaked in the first day but quickly declined and diminished after 4 days (Figure 4A). In addition, we treated NSG mice with lower doses of EGFRvIII-DBTE and observed that the day 14 sera of the mice treated with as low as a 10-μg dose induced significant cytotoxicity against U87vIII cells (Figure 4B). These results illustrate that a single injection of EGFRvIII-DBTE can produce durable and potent *in vivo* expression, which is not observed following a single dose of protein BTE infusion and is dose-sparing.

Heterotopic model of GBM

To evaluate *in vivo* cytotoxicity of EGFRvIII-DBTE, we conducted a GBM challenge in NSG mice by injecting U87vIII cells subcutaneously in the right flank. At day 8 when tumor size had grown to 50 mm^3, the mice were given an IM injection of 100 μg EGFRvIII-DBTE or pVAX1 in the TA muscle followed by EP and an i.p. injection of primary human T cells. Seven days later, we treated mice with another injection of DNA/EP and continued to monitor the tumor sizes over time (Figure 5A). We observed that all five of five mice treated with EGFRvIII-DBTE demonstrated tumor regression, with four animals clearing the challenge, whereas zero of five mice treated with pVAX1 controlled their tumor growth (Figures 5B and 5C).

EGFRvIII-DBTE clears tumor burden in an orthotopic animal model of GBM

To evaluate the efficacy of EGFRvIII-DBTE in an orthotopic model, we conducted an intracranial GBM challenge in NSG mice by injecting 1×10^5 U87vIII-luc cells in the right hemisphere of NSG mice. At day 6, we treated the mice with EGFRvIII-DBTE, HER2-DBTE, or pVAX1 in the TA muscle followed by EP. At day 7, mice received an i.p. injection of primary human T cells (Figure 6A). Tumor burden was monitored by IVIS Spectrum using *in vivo*-grade luciferin. We observed that 10 of 10 mice treated with EGFRvIII-DBTE exhibited tumor clearance. None of 10 mice treated with pVAX1 or 10 mice treated with HER2-DBTE targeting irrelevant antigen in this model showed tumor regression (Figures 6B–6D). All 20 of the control animals succumbed to the challenge, demonstrating the specificity of the

Figure 3. EGFRvIII-DBTE induces T cell activation

(A) Fluorescent images of U87vIII cells in a tumor-killing assay upon addition of mouse sera treated with EGFRvIII-DBTE or pVAX1. Day 14 sera were used. Target cells are shown in green. CD69 activation is shown in red. Caspase-3 induction is shown in blue. (B–D) Quantified GFP^+ cell counts, CD69 activation, and caspase-3 induction in the tumor-killing assay. Flow cytometry data showing (E) IFN-γ, TNF-α, IL-2, and (F) CD107a responses in $CD4^+$ T cells and $CD8^+$ T cells in a 24-h tumor-killing assay. (G) Tumor-killing assay with $CD4^+$ T cells and/or $CD8^+$ T cells in the presence of EGFRvIII-DBTE. (H) Fluorescent images of the target cells at 0-, 6-, and 24-h time points.

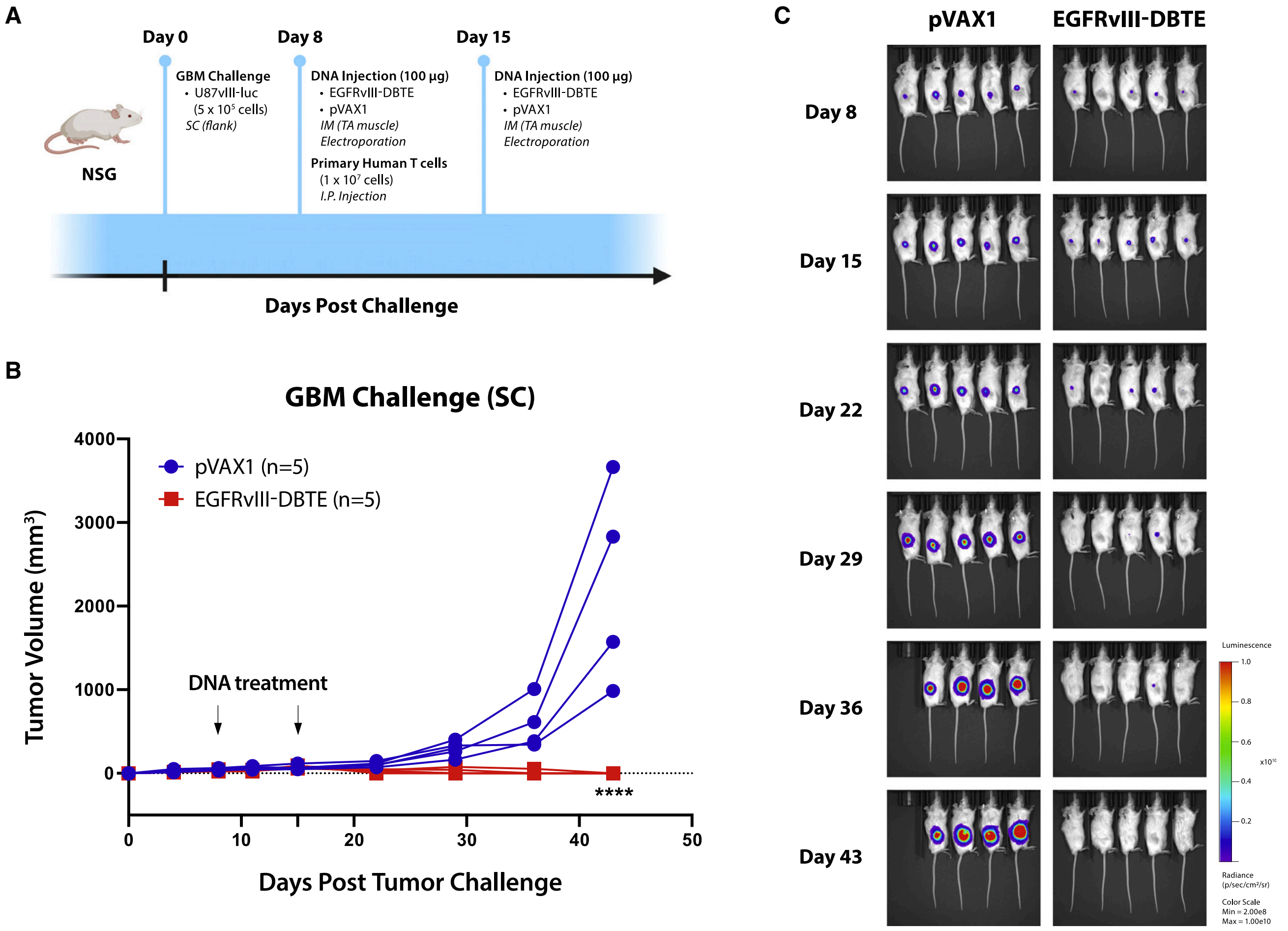

Figure 5. Heterotopic GBM challenge

(A) A scheme of heterotopic GBM challenge in NSG mice. (B) Tumor volume in the challenge plotted over time. (C) IVIS bioluminescent images of tumor burden in the challenged mice.

EGFRvIII-DBTE. This study in NSG mice did not continue beyond approximately 28 to 34 days due to onset of chronic graft versus host disease in the surviving animals, as is described for the used model.[19] Cryosections of the mouse brains were collected at the endpoints and examined by confocal microscopy for EGFRvIII expression in the tumor region of the brain. We observed that the brains of EGFRvIII-DBTE-treated mice showed clearance of tumor burden as well as EGFRvIII expression, neither of which was observed in the brains of pVAX1-treated mice (Figure 6E).

EGFRvIII$^+$/HER2$^+$ heterogeneous model of GBM

A significant challenge for GBM immunotherapy remains the heterogeneity of antigen expression, which permits tumor escape in single-agent immunotherapeutic approaches.[5,6,11] A strategy for overcoming this issue could be co-delivery of multiple DBTEs targeting additional antigens. Here we targeted both EGFRvIII and HER2, which are expressed in up to 30% and 80% of GBM cases, respectively.[3,16] In this set of studies, we used EGFRvIII-DBTE in conjunction with previously described HER2-DBTE, which showed efficacy in a HER2-expressing tumor model.[15] To develop an EGFRvIII$^+$/HER2$^+$ heterogeneous model of GBM, we chose U87vIII (EGFRvIII$^+$/HER2$^-$) and U251 cells (EGFRvIII$^-$/HER2$^+$) (Figure S1B).

U87vIII and U251 cells were plated together in the same well in a 1:1 ratio mixture. Sera from mice treated with EGFRvIII-DBTE, HER2-DBTE, or combination of the two DBTEs was then added to the tumor cells along with primary T cells. After a 48-h incubation, the mice co-treated with both EGFRvIII-DBTE and HER2-DBTE exhibited enhanced cytotoxicity against heterogeneous tumor cells compared with sera of the mice treated with single DBTE (Figure 7A). We also observed that co-administration of the DBTEs did not impair killing of U87vIII cells when compared with EGFRvIII-DBTE or U251 cells when compared with HER2-DBTE, indicating co-delivery of the two DBTEs does not interfere with the expression and the tumor-killing capabilities of one another. In the heterogeneous

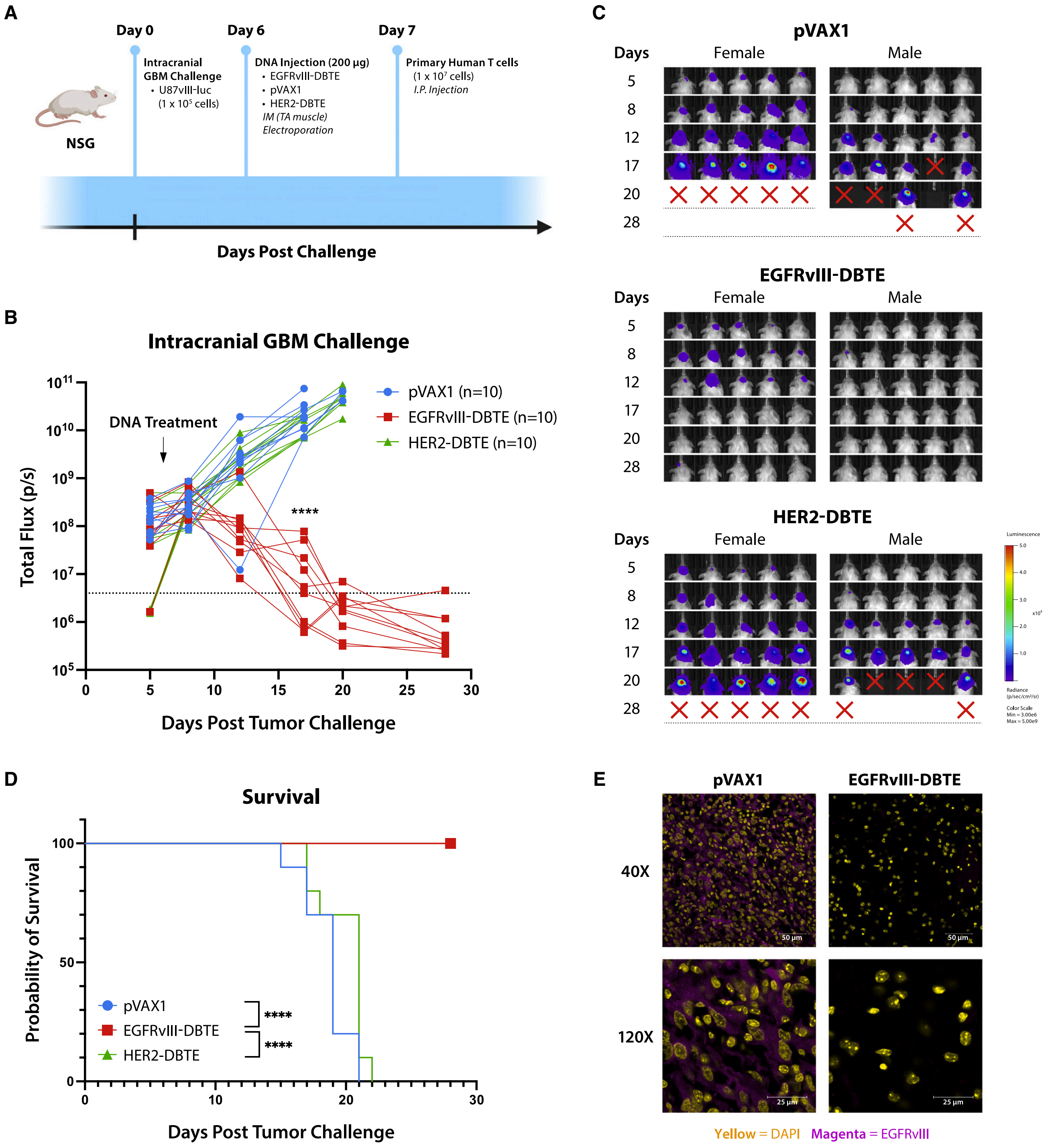

Figure 6. Intracerebral GBM challenge

(A) A scheme of intracranial GBM challenge in NSG mice. (B) Tumor burden of the challenged mice measured by IVIS. (C) IVIS images of the challenged mice. (D) Survival of the challenged mice. (E) Representative confocal images of brain sections of the challenged NSG mice at the endpoint of the study. EGFRvIII expression is shown in magenta. Nuclei are shown in yellow.

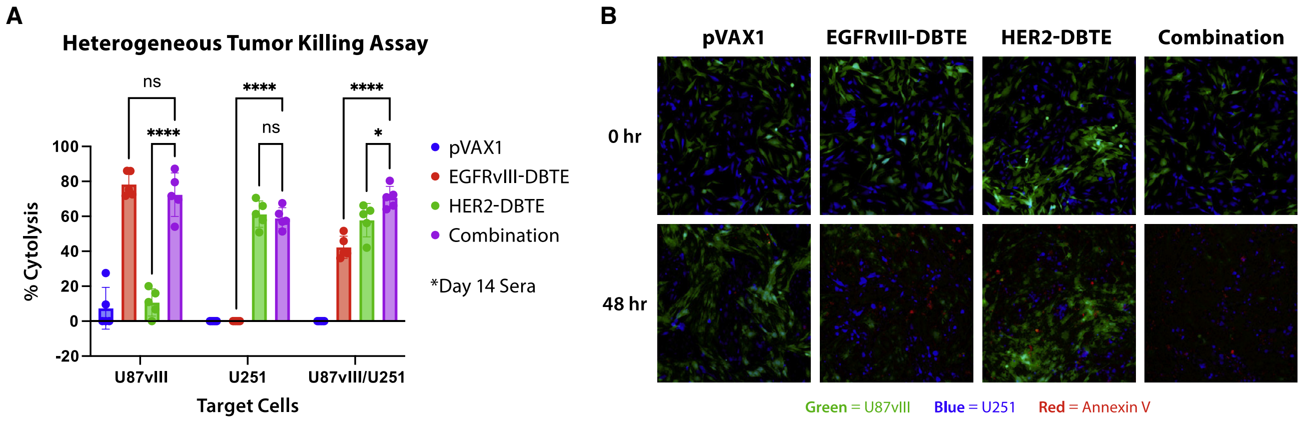

Figure 7. EGFRvIII$^+$/HER2$^+$ heterogeneous model of GBM

(A) T cell-mediated cytotoxicity assay against U87vIII cells (EGFRvIII$^+$) and/or U251 cells (HER2$^+$) using NSG mice treated with EGFRvIII-DBTE and/or HER2-DBTE. (B) Fluorescent images of the heterogeneous tumor mixture (U87vIII/U251) in a 48-h tumor-killing assay.

tumor-killing assay, we dyed U251 cells with a cell-trace blue dye and observed by fluorescent microscopy that the mouse sera co-treated with two DBTEs induced apoptosis in both U87vIII (GFP) and U251 (blue) cell populations (Figure 7B).

Co-delivery of EGFRvIII-DBTE and HER2-DBTE enhanced tumor regression and improved survival in an orthotopic animal model of heterogeneous GBM

To further investigate the efficacy of a combined treatment of EGFRvIII-DBTE and HER2-DBTE, we developed and conducted an intracranial challenge of heterogeneous GBM in NSG mice. In this model, we implanted 5×10^4 U87vIII-luc cells and 5×10^4 U251-luc cells in a single injection in the right hemisphere of NSG mice, with five female and five male mice per group. On day 6 of challenge, we treated animals with a single 200-μg dose of pVAX1, EGFRvIII-DBTE, HER2-DBTE, or both DBTEs delivered in separate sites. All mice were given an i.p. injection of 1×10^7 primary human T cells the following day (Figure 8A). Tumor burden was monitored by IVIS Spectrum using *in vivo*-grade luciferin. We observed enhanced tumor regression and survival in the group that received combined treatment of the two DBTEs over the groups that received single DBTE treatments (Figures 8B–8F). In the pVAX1-treated group, uncontrolled, aggressive tumor growths were observed in 10 of 10 mice (Figure 8B). In the EGFRvIII-DBTE-treated group, seven of 10 mice showed moderate tumor control initially and two mice lost tumor control soon after treatment, while one mouse succumbed to challenge after initial tumor escape (Figure 8C). In the HER2-DBTE-treated group, one mouse showed tumor regression while nine mice lost tumor control (Figure 8D). In the combined treatment group, eight of 10 mice showed complete tumor regression while two mice exhibited tumor escape (Figure 8E). At study completion on day 34, 80% of the mice that received both DBTEs survived the challenge, whereas 20% in the EGFRvIII-DBTE group, 10% in HER2-DBTE group, and 0% in pVAX1 group survived the heterogeneous GBM challenge (Figure 8F). This study was limited to 34 days due to onset of chronic graft versus host disease in the surviving animals.[19]

At the endpoints of the study, cryosections of the brains were collected and examined for EGFRvIII and HER2 expression in the tumor region. By fluorescent confocal microscopy, we observed that the mice that received combination treatment cleared the tumor burden and demonstrated loss of both EGFRvIII and HER2 expressing cells, while EGFRvIII-DBTE-treated mice showed HER2 expression and conversely HER2-DBTE-treated mice showed continued EGFRvIII expression in the tumor sections (Figure 8G). These data strongly support that the co-treatment of EGFRvIII-DBTE and HER2-DBTE result in improved efficacy in controlling heterogeneous GBM tumors through reduced heterogeneous tumor escape and illustrate the non-competitive nature of this combination approach.

DISCUSSION

In this article, we developed a new EGFRvIII-DBTE for study in therapeutic models of GBM, exploring its anti-tumor cytotoxicity, specificity, T cell activation, *in vivo* pharmacokinetics, and impact in GBM challenge models. This treatment exhibited durable *in vivo* expression of EGFRvIII-DBTE over 15 weeks, with killing activity and continued ability to lower and clear tumor burden in all treated animals in an intracranial challenge of GBM after a single dose. We next studied a dual tumor targeting approach combining EGFRvIII-DBTE and HER2-DBTE treatments in a heterogeneous challenge model. In this combination approach, treatment with two DBTEs exhibited impressive control of heterogeneous GBM tumors and mitigated immune escape in 80% of the challenged mice.

We also studied the detailed characterization of EGFRvIII in impact on tumor. The EGFRvIII-DBTE exhibited specific binding activities and cytotoxicity against EGFRvIII-expressing GBM cells (Figures 1C–1E and 2A), while not binding to wild-type EGFR (Figure S2) or inducing anti-tumor cytotoxicity in the absence of either

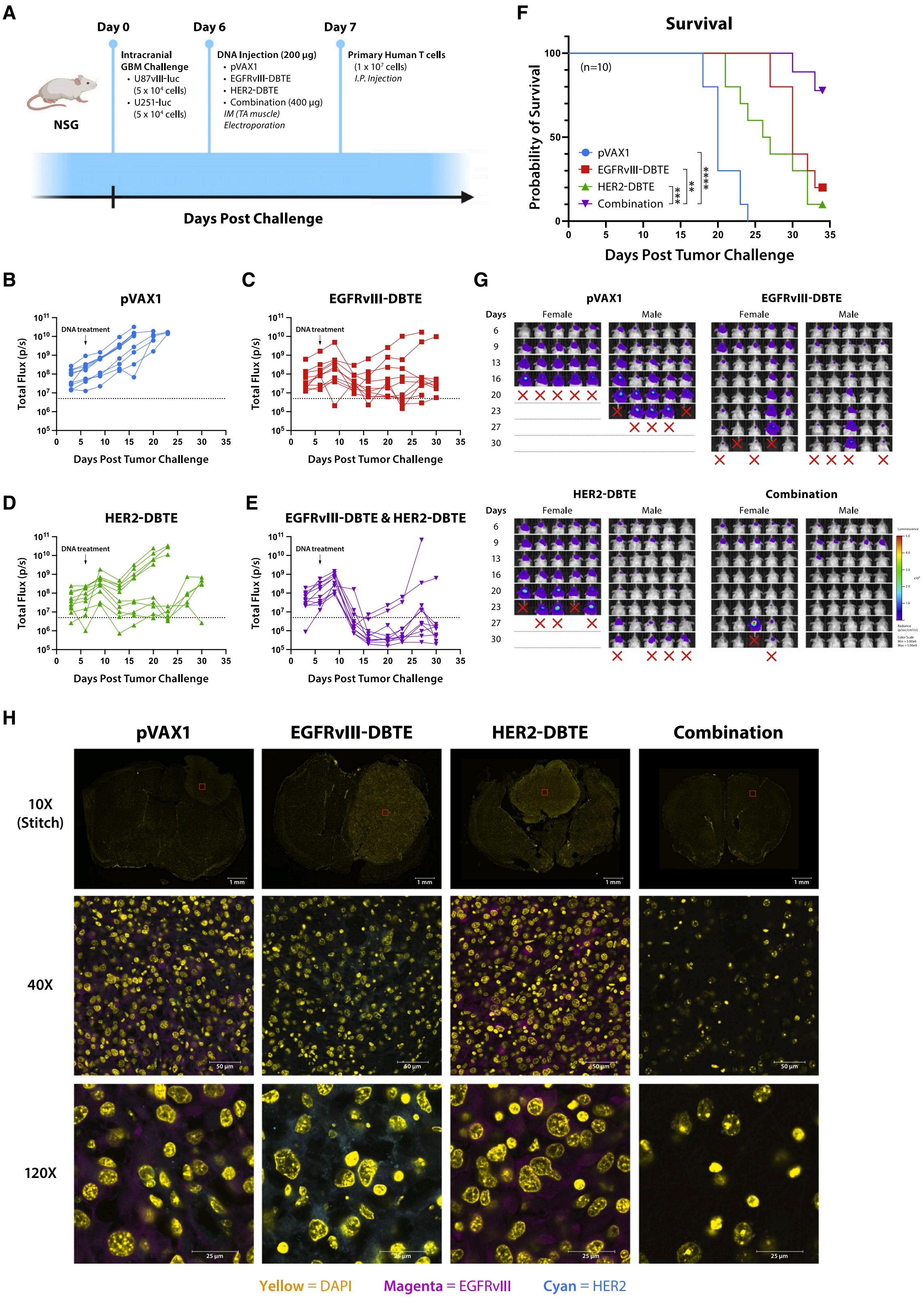

(legend on next page)

targets, EGFRvIII or CD3 (Figures 2B and 2C). Safety and toxicity are of concern in cancer immunotherapeutic agents in development. These data support the specificity of EGFRvIII-DBTE, reducing the potential of off-target toxicity. Also, the DNA vector itself has been reported to show low intrinsic immunogenicity and high safety profile in clinical trials.[20,21]

We characterized the T cell responses driven by the EGFRvIII-DBTE in an activation assay against U87vIII cells and observed that EGFRvIII-DBTE induced CD69 activation in T cells (Figure 3A) and anti-tumor cytokine release in both $CD8^+$ and $CD4^+$ T cell populations (Figures 3E and 3F). One particularly important aspect was that $CD4^+$ T cells also exhibited clear, but lower CD107a activation, supporting that cytolytic activities of $CD4^+$ T cells are also induced by EGFRvIII-DBTE (Figure 3F).[22] We analyzed $CD4^+$ T cells in a tumor-killing assay and observed that EGFRvIII-DBTE induced anti-tumor cytotoxicity by isolated $CD4^+$ T cells at an E:T ratio of 20:1 (Figures 3G and 3H). In the tumor microenvironment for immune-cold tumors like GBM, $CD8^+$ T cells often enter an inactivated state and instead $CD4^+$ T cell tumor infiltration is observed. A high level of $CD4^+$ tumor-infiltrating lymphocytes (TILs) with a low level of $CD8^+$ TILs is associated with poor prognoses, which has been reported in glioma patients.[23] The role of $CD4^+$ T cells in cancer was thought to be primarily in priming immune response for CTLs. However, recent emerging evidence suggests that under some conditions, $CD4^+$ T cells can participate in cytolytic activities against tumors.[24] Our results expand on this work demonstrating that the stimulation using EGFRvIII-DBTE can induce $CD4^+$ T cells to participate directly to kill target GBM and likely can be an important tool to recruit effector $CD4^+$ T cells to target these tumors.

It has been suggested that the GBM microenvironment may impose challenges in *in vivo* delivery due to its immunosuppressive properties and the presence of the blood-brain barrier (BBB). However, recent reports suggest that BBB is impaired in GBM, which likely allows for increased passive diffusion, and the bioavailability of the peripherally delivered drugs in the brain remains of concern.[25,26] Thus, agents that offer efficacy at low concentrations are likely important for treatment of GBM. In a killing assay, we observed that EGFRvIII-DBTE induced potent cytotoxicity against U87vIII cells with a dose with EC_{50} of 41.5 pM (Figure 2D) and function at a low E:T ratio of 1:1 (Figure 2E). This engineered EGFRvIII-DBTE is a potent T cell engager that maintains function in the modeled GBM microenvironment studied here. We demonstrated potency in the intracranial challenge, observing that the EGFRvIII-DBTE coordinates T cells to the target and reduces tumor load in the challenge model, resulting in complete clearance of tumor in all treated animals by a single injection (Figures 6B–6D).

In *in vivo* pharmacokinetics study, a single injection of EGFRvIII-DBTE exerted tumor-killing activity lasting more than 15 weeks (Figure 4A). In contrast, a single injection of a recombinant EGFRvIIIxCD3 delivered peripherally exhibited cytotoxicity that lasted for just 4 days. Due to their short serum half-life, a protein-based EGFRvIII-targeted BTE required multiple doses over 16 consecutive days to exert significant tumor regression and show 72% survival in an animal challenge in a recent study.[14] However, in the intracranial challenge study described here, we observed that a single injection of the EGFRvIII-DBTE exhibited complete tumor clearance with 100% (10 of 10 animals) survival (Figures 6B–6D), providing a simpler and potentially important additional treatment tool. It is possible that such genetic-based biologics could be dose- and cost-sparing.[27] DBTEs may allow for tumor targeting and control for longer periods of time, potentially lowering treatment cost for patients.

The animal model used in which NSG mice are repopulated with primary human T cells is well established and a widely used model for development of bivalents and CAR-Ts, due to anti-drug antibody (ADA) responses in immunocompetent animal models.[28–30] ADA response is a significant challenge for antibody therapies in immune-competent models and does appear in the clinic at a detectable level. Humanized antibodies such as Blinatumomab, an FDA-approved BTE, have lower immunogenicity than mouse antibodies in human subjects, reporting <1% ADA responses in treated patients.[31,32] EGFRvIII-DBTE and HER2-DBTE described in this article are humanized antibodies, lowering the risk of ADA responses in humans. *In vivo* delivery by electroporation alone does not induce an ADA response, as we previously reported that a DNA/EP delivery in mice of a species-matched DNA-encoded monoclonal antibody resulted in a durable antibody expression over 10 weeks without detectable ADA responses.[33]

A challenge for immunotherapy for GBM cancer remains antigen heterogeneity. GBM displays various degrees of antigens such as EGFRvIII and HER2 in a heterogeneous manner.[7,8] Immunotherapies such as CAR-T and peptide vaccine, which targeted EGFRvIII alone, have not yet demonstrated significant clinical benefits in GBM patients thus far.[5,6] In single antigen-targeting therapies, tumor cells that are not recognized by the therapy likely use immune escape mechanisms by mutation of tumor antigen and selective survival of antigen-negative tumor subpopulations. Strategies that target multiple tumor antigens will likely help mitigate immune escape in this context. Tri-specific antibodies for dual tumor antigen targeting have been previously reported.[34,35] However, these approaches used tumor models in which two antigens are homogeneously expressed and failed to address the impact of biologics in suppression of tumor

Figure 8. Co-delivery of EGFRvIII-DBTE and HER2-DBTE in heterogeneous GBM challenge

(A) A scheme of heterogeneous orthotopic GBM challenge in NSG mice wherein a mixture of U87vIII cells and U251 cells were inoculated in the brain. (B–E) Tumor burden of the challenged mice that received a treatment of (B) pVAX1, (C) EGFRvIII-DBTE, (D) HER2-DBTE, or (E) both EGFRvIII-DBTE and HER2-DBTE. (F) Survival of the challenged NSG mice. (G) IVIS images of the challenged mice. (H) Representative confocal images of the brain sections of the challenged mice at the endpoints of the study. EGFRvIII expression is shown in magenta. HER2 expression is shown in cyan. Nuclei are shown in yellow.

escape. Here, we described a co-delivery of multiple bispecific biologics in a heterogeneous *in vivo* tumor model wherein NSG mice are orthotopically challenged with a mixture of EGFRvIII-expressing tumor cells (U87vIII) and HER2-expressing tumor cells (U251). We observed that sera from animals that received co-treatment of EGFRvIII-DBTE and a HER2-DBTE induced cytotoxicity against both EGFRvIII$^+$ and HER2$^+$ tumor populations (Figures 7A and 7B). In an intracranial heterogeneous GBM challenge, a single injection of the combined treatment of these DBTEs exerted enhanced tumor regression and improved survival as compared with single DBTE treatments or controls (Figures 8B–8F). Eighty percent of tumor-bearing mice showed persistent tumor control and clearance if administered both DBTEs at the same time. This was not observed in mice treated with single DBTEs (Figure 8F), supporting that the combined treatment with two DBTEs targeting two different antigens enhanced tumor suppression and improved morbidity in challenge. Examination of brain sections collected at the endpoints for each mouse revealed antigen escape in mice that received a single-agent therapy, but not in mice that received the combined treatment of both DBTEs (Figure 8G). The two DBTEs effectively controlled tumor growth and mitigated antigen escape in the heterogeneous GBM challenge.

In conclusion, the simplicity in production and delivery of DBTEs suggests the importance of studying combination approaches targeting multiple tumor antigens. GBM expresses many other antigens that are receiving attention, such as IL13Ra2 and EphA2. A combination approach as we illustrate here could be expanded to target additional antigens, potentially further improving tumor control and advancing patient outcomes. Broadening treatment options for GBM and other cancer patients with combination therapies, potentially providing a personalized combination of DBTEs based on antigen expression profile of each patient, deserve additional study.

MATERIALS AND METHODS

Animals and cell lines

Male and female NSG mice were obtained from the Wistar Institute Animal Facility and used in *in vivo* expression studies and GBM challenge models. All animals were housed at the Wistar Institute animal facilities and given free access to food and water in groups of five animals per cage. Animal experiments were approved by the Institutional Animal Care and Use Committee at The Wistar Institute (protocol 201,401).

EGFRvIII-expressing U87-MG (U87vIII) cell line was generated by sequentially transducing U87-MG tumor cells (HTB-14, ATCC) with firefly luciferase lentivirus (PLV-10003, Cellomics Technology) and virus-containing media of Phoenix-AMPHO cells (CRL-3213, ATCC) transfected with pBMN-I-GFP embedding human EGFRvIII, which was generated by Genscript. Transduced cells were sorted by GFP expression and EGFRvIII expression was validated by anti-human EGFRvIII flow antibody (NBP2-50599, Novus Biologicals). U251-luc cell line was generated by transduction of U251-MG tumor cells (09,063,001, Millipore Sigma) with firefly luciferase lentiviral vector (PLV-10003, Cellomics Technology). DK-MG cells were obtained from Amsbio (CL 01008-CLTH). Tumor cells were kept in low passage number, cultured in MEM (or RPMI1640 for DK-MG) containing 10% heat-inactivated FBS and 100 U/mL penicillin/streptomycin, at 37°C in a 5% CO_2 incubator.

Expi293F cell line (Thermo Fisher Scientific, A14527) was used for *in vitro* expression studies. Expi293F cells were cultured in Expi293 expression medium (A1435101, Thermo Fisher Scientific) and kept in suspension by an orbital shaker, at 37°C in an 8% CO_2 incubator.

Primary human T cells were obtained from healthy donors at Human Immunology Core at University of Pennsylvania by negative selection using RosetteSep Human T cell isolation kit (Stemcell, #15061). T cells were cultured in RPMI1640 containing 10% heat-inactivated FBS and 100 U/mL penicillin/streptomycin, at 37°C in a 5% CO_2 incubator. In GBM challenge studies, T cells were activated and expanded with T cell activation/expansion kit (130-091-441, Miltenyi Biotec) and recombinant IL-2 (130-097-745, Miltenyi), following the manufacturer's protocol.

Design of EGFRvIII-DBTE and HER2-DBTE

We designed EGFRvIII-DBTE by encoding a codon-optimized sequence of EGFRvIII-binding scFv[17] fused with humanized CD3-binding scFv (clone UCHT-1) by a GS linker. Human IgE leader sequence was added to the N terminus of the construct. Altogether, the construct was subcloned into a modified pVAX1 expression vector. Previously described HER2-DBTE[15] is composed of HER-binding scFv fused with CD3-binding scFv.

In vitro expression of DBTEs

For *in vitro* expression of DBTEs, Expi293F cells were transfected with DBTE constructs by using ExpiFectamine 293 transfection kit (A14524, Thermo Fisher Scientific), following the manufacturer's protocol. Supernatants were collected at day 5 of transfection.

Western blot

The total protein concentration of the supernatant samples from DBTE-transfected Expi293F cells were quantified using a bicinchoninic acid assay (Pierce, Thermo Fisher Scientific). Ten micrograms of supernatant samples were loaded on a 4%–12% Bis-Tris SDS-PAGE gel (NuPAGE, Thermo Fisher Scientific). The gel was transferred to a PVDF membrane using the iBlot 2 system (Thermo Fisher Scientific). The membrane was blocked in Intercept (PBS) blocking buffer (Licor) and then probed with a donkey anti-human IgG H + L secondary antibody (Licor) diluted 1:15,000 in Intercept T20 (PBS) antibody diluent (Licor). The membrane was scanned with Odyssey CLx imaging system (Licor). Western blotting was performed three times.

Quantitative ELISA

Ninety-six-well plates (Fisher) were coated with anti-human F(ab')2 antibody (Novus Bio) and incubated overnight at 4°C. The plates were blocked in PBS, 10% FBS, for 1 h and the diluted samples and

standards were added for 1 h. Then they were probed with 1:5,000 anti-human F(ab')2 antibody, horseradish peroxidase-conjugated (Jacksonimmuno Research) for 1 h. The plates were developed using TMB solution (ThermoFisher) for 10 min and stopped using 2N H_2SO_4 solution. The optical densities were measured at 450 nm using plate scanner (BioTek Synergy 2). The concentration of samples was determined based on the standard curve (4-parameter sigmoidal) using purified bispecific antibodies as standards. The purified bispecific antibodies were generated by Genscript by CHO transfection followed by purification using 6X HisTag at N terminus, which then was removed by protease. Quantitative ELISA experiments were performed with three replicates of each sample and standard.

T cell-mediated cytotoxicity assay

U87vIII cells or U251-luc cells were plated on 96-well E-plate (ACEA biosciences) at 1×10^4 cells/well in 100 μL RPMI 1640 medium containing 10% FBS (R10) and incubated at 37°C overnight. Pre-treatment cell viability of the target cells was monitored by xCelligence RTCA eSight machine for 18 to 20 h. Primary human T cells were rested at 37°C overnight in R10 and added to the target cells at various effector to target ratios together with DBTE-containing supernatant (10 ng/mL) or mouse serum (diluted 1:10) in a total volume of 100 μL. The cell viability was monitored with xCelligence RTCA eSight for 48 h. The cell viability of each assay well was normalized to the last cell index of pre-treatment incubation. Percent cytolysis was plotted as the percent difference of cell indices from the baseline (target cells with T cells only) at each time point. For fluorescent imaging, human CD69 antibody conjugated with Alexa Fluor 647 (FAB23591R, R&D Systems) and caspase-3 blue dye (SCT102, Millipore Sigma) were added at 10 μg/mL to the wells upon addition of effector cells. Bright field images and fluorescent (green, blue, red) images were taken with xCelligence RTCA eSight. T cell-mediated cytotoxicity assays were performed with three replicates for *in vitro* samples and five replicates for *in vivo* samples.

Flow cytometry

U87vIII cells were plated on a 96-well plate (ThermoFisher) at 1×10^4 cells/well in 100 μL RPMI 1640 medium containing 10% FBS (R10) and incubated at 37°C overnight.

Primary human T cells were rested overnight in a 37°C incubator and 5% CO_2 and added to the target cells together with DBTE-containing supernatant, normalized at 10 ng/mL, in a total volume of 100 μL. A 1X Protein transport inhibitor cocktail (eBioSciences, 00-4980-03) and CD107a antibody conjugated to PE-Cy7 (clone H4A3, Biolegend) diluted 1:100 were added to the wells. After 24-h incubation in a 37°C incubator and 5% CO_2, T cells were collected and washed with PBS. T cells were first incubated with Live/Dead viability stain (Zombie Yellow, Biolegend) diluted 1:1,000 in PBS for 10 min, and then CD4 conjugated to BV510 (clone OKT4, Biolegend) and CD8 conjugated to APC-Cy7 (clone SK1, Biolegend) diluted 1:100 in PBS with 1% FBS for 30 min. Cells were then fixed and permeabilized using Cytofix/Cytoperm reagents (554,714, BD Biosciences). Further intracellular staining was performed using IFN-γ conjugated to AF700 (clone B27, Biolegend), TNF-α conjugated to AF488 (clone MAb11, Biolegend), and IL-2 conjugated to PerCP/Cy5.5 (clone MQ1-17H12, Biolegend) diluted 1:100 in Perm/Wash buffer (554,723, BD Biosciences) for 1 h. Single stain and fluorescence minus one (FMO) controls were included for gating. Samples were analyzed using a BD LSR II flow cytometer and data were analyzed using FlowJo 10 software. Boolean gating was performed on T cell populations specifically secreting IFN-γ, TNF-α, and/or IL-2. Flow cytometry experiments were performed with three replicates of each sample.

DBTE treatment in mice

For *in vivo* expression studies and tumor challenge studies, mice received intramuscular injections (100 μg/site DNA plasmid) in TA muscles of EGFRvIII-DBTE, HER2-DBTE, or pVAX1 DNA plasmid co-formulated with hyaluronidase (200 U/L, Sigma Aldrich, Saint Louis, MO), followed by electroporation (IM-EP) using the CELLECTRA 3P adaptive constant current device (Inovio Pharmaceuticals, Plymouth Meeting, PA). Serum was collected longitudinally to monitor *in vivo* expression.

Mouse xenograft studies

In the heterotopic GBM challenge study, five female NSG mice in each group were inoculated with GBM tumors via subcutaneous injection of U87vIII (5×10^5 cells in 100 μL of PBS) in the right flank. Tumor size was measured longitudinally with a digital caliper and the volume was calculated using the formula, $V = (W^2 \times L)/2$. When the tumor size reached 50 mm^3, the mice received an intramuscular (IM) injection of DNA treatment in the TA and an i.p. injection of 1×10^7 primary human T cells in 100 μL PBS. A second dose of DNA treatment was administered 7 days later. Tumors were scanned with IVIS Spectrum following i.p. injection of *in vivo*-grade luciferin (Promega). The mice were euthanized when tumor size reached 2,000 mm^3.

In the intracranial GBM challenge studies, five male and five female NSG mice in each group received intracranial injection of 1×10^5 tumor cells into the striatum. Mice were anesthetized with a cocktail of ketamine (Vedco, St. Joseph, MO, USA) and xylazine (Akorn Animal Health, Lake Forest, IL, USA). Skull was trepanned with a drill 1 mm posterior to the bregma and 2 mm lateral to the midline. At 2.5 mm in depth, a 2-μL injection of 1×10^5 tumor cells was inoculated over 2 min using a stereotactic frame and automatic syringe pump (Stoelting Co., Wood Dale, IL, USA). The syringe was withdrawn slowly (0.5 mm/min) and then the incision was sutured (Ethicon Inc., Somerville, NJ, USA). Mice received antibiotic ointment over the incision and a subcutaneous injection of buprenorphine analgesic. Then mice were monitored for adverse responses and weight loss. Mice that lost 20% of initial weight or had symptoms of graft versus host disease were euthanized in CO_2 chamber. Randomization was performed prior to DNA treatments.

Fluorescent immunohistochemical images of murine brain sections

At endpoint of the studies, mouse brains were harvested and fixed by sequentially incubating in 10% formalin (Millipore Sigma,

USA), 15% and 30% sucrose solutions. The specimens were embedded in O.C.T. compound and frozen rapidly in dry ice. Ten-micron coronal sections of the brain specimens were performed by the Histotechnology Core at the Wistar Institute. The frozen section slides were blocked with 5% normal goat serum in PBS and stained with 10 μg/mL anti-EGFRvIII murine antibody conjugated to AF647 (clone DH8.3; Novus Biologicals, USA) and 10 μg/mL anti-HER2 murine antibodies conjugated to AF555 (clone EP1045Y; Abcam, USA) and DAPI. The fluorescent confocal images of the sections were captured by the Imaging Facility at the Wistar Institute using Leica TCS SP8 confocal microscope. Section samples were blinded to the Imaging Facility.

Statistics

The data were graphed and statistical analyses performed using GraphPad Prism 9.0 software (La Jolla, CA). Statistical comparisons included a two-way ANOVA, with correction for multiple comparisons, which compares groups within each time point (simple effects within rows). Survival data were represented by a Kaplan-Meier survival curve and significance was calculated using a log rank test between each group. In all experiments, samples with a p value < 0.05 were considered statistically significant. The line graphs represent individual animals, where indicated. Scatterplots display individual animals, the mean value, and error bars represent the standard deviation.

DATA AVAILABILITY

All data are available in the main text or supplemental information.

SUPPLEMENTAL INFORMATION

Supplemental information can be found online at https://doi.org/10.1016/j.omto.2023.02.004.

ACKNOWLEDGMENTS

We thank the Animal Facility at the Wistar Institute for providing care for all animals in the study, the Flow Core at the Wistar Institute for assistance in flow experiments, the Histotechnology Facility at the Wistar Institute for providing cryosections of animal tissues, the Imaging Facility at the Wistar Institute for assistance in confocal microscopy, and the Human Immunology Core at the University of Pennsylvania for providing primary human T cells. The graphical abstract and some illustrative figures were created with BioRender (BioRender.com). This research was funded by a sponsored research agreement from INOVIO Pharmaceuticals. D.B.W. is supported by the W.W. Smith Charitable Trust Professorship in Cancer Research, which covered aspects of the work.

AUTHOR CONTRIBUTIONS

D.H.P., A.P.P., A.P., and D.B.W. conceptualized and designed experiments. D.H.P. and A.P.P. engineered plasmid DBTE constructs. D.H.P., K.L., P.B., X.Z., J.C., D.B., E.N.G., R.P.O., A.R.A., and A.K. performed experiments and analyzed data. D.H.P. contributed substantially to writing. All authors contributed to revision of the manuscript.

DECLARATION OF INTERESTS

D.B.W. has received grant funding, participates in industry collaborations, has received speaking honoraria, and has received fees for consulting, including serving on scientific review committees. Remunerations received by D.B.W. include direct payments and equity/options. D.B.W. also discloses the following associations with commercial partners: Geneos (consultant/advisory board), AstraZeneca (advisory board, speaker), Inovio (board of directors, consultant), Sanofi (advisory board), BBI (advisory board), Pfizer (advisory board), Flagship (consultant), and Advaccine (consultant).

REFERENCES

1. Taylor, O.G., Brzozowski, J.S., and Skelding, K.A. (2019). Glioblastoma multiforme: an overview of emerging therapeutic targets. Front. Oncol. *9*, 963.
2. Bausart, M., Préat, V., and Malfanti, A. (2022). Immunotherapy for glioblastoma: the promise of combination strategies. J. Exp. Clin. Cancer Res. *41*, 35.
3. Felsberg, J., Hentschel, B., Kaulich, K., Gramatzki, D., Zacher, A., Malzkorn, B., Kamp, M., Sabel, M., Simon, M., Westphal, M., et al. (2017). Epidermal growth factor receptor variant III (EGFRvIII) positivity in EGFR-amplified glioblastomas: prognostic role and comparison between primary and recurrent tumors. Clin. Cancer Res. *23*, 6846–6855.
4. Padfield, E., Ellis, H.P., and Kurian, K.M. (2015). Current therapeutic advances targeting EGFR and EGFRvIII in glioblastoma. Front. Oncol. *5*, 5.
5. O'Rourke, D.M., Nasrallah, M.P., Desai, A., Melenhorst, J.J., Mansfield, K., Morrissette, J.J.D., Martinez-Lage, M., Brem, S., Maloney, E., Shen, A., et al. (2017). A single dose of peripherally infused EGFRvIII-directed CAR T cells mediates antigen loss and induces adaptive resistance in patients with recurrent glioblastoma. Sci. Transl. Med. *9*, eaaa0984.
6. Schuster, J., Lai, R.K., Recht, L.D., Reardon, D.A., Paleologos, N.A., Groves, M.D., Mrugala, M.M., Jensen, R., Baehring, J.M., Sloan, A., et al. (2015). A phase II, multicenter trial of rindopepimut (CDX-110) in newly diagnosed glioblastoma: the ACT III study. Neuro. Oncol. *17*, 854–861.
7. Saikali, S., Avril, T., Collet, B., Hamlat, A., Bansard, J.Y., Drenou, B., Guegan, Y., and Quillien, V. (2007). Expression of nine tumour antigens in a series of human glioblastoma multiforme: interest of EGFRvIII, IL-13Ralpha2, gp100 and TRP-2 for immunotherapy. J. Neurooncol. *81*, 139–148.
8. Liu, G., Ying, H., Zeng, G., Wheeler, C.J., Black, K.L., and Yu, J.S. (2004). gp100, and MAGE-1 are expressed in human glioblastoma and recognized by cytotoxic T cells. Cancer Res. *64*, 4980–4986.
9. Nagaraj, S., Gupta, K., Pisarev, V., Kinarsky, L., Sherman, S., Kang, L., Herber, D.L., Schneck, J., and Gabrilovich, D.I. (2007). Altered recognition of antigen is a mechanism of CD8+ T cell tolerance in cancer. Nat. Med. *13*, 828–835.
10. Furnari, F.B., Cloughesy, T.F., Cavenee, W.K., and Mischel, P.S. (2015). Heterogeneity of epidermal growth factor receptor signalling networks in glioblastoma. Nat. Rev. Cancer *15*, 302–310.
11. Vinay, D.S., Ryan, E.P., Pawelec, G., Talib, W.H., Stagg, J., Elkord, E., Lichtor, T., Decker, W.K., Whelan, R.L., Kumara, H.M.C.S., et al. (2015). Immune evasion in cancer: mechanistic basis and therapeutic strategies. Semin. Cancer Biol. *35*, S185–S198.
12. Goebeler, M.E., and Bargou, R.C. (2020). T cell-engaging therapies - BiTEs and beyond. Nat. Rev. Clin. Oncol. *17*, 418–434.
13. Zhou, S., Liu, M., Ren, F., Meng, X., and Yu, J. (2021). The landscape of bispecific T cell engager in cancer treatment. Biomark. Res. *9*, 38.
14. Sternjak, A., Lee, F., Thomas, O., Balazs, M., Wahl, J., Lorenczewski, G., Ullrich, I., Muenz, M., Rattel, B., Bailis, J.M., and Friedrich, M. (2021). Preclinical assessment of AMG 596, a bispecific T-cell engager (BiTE) immunotherapy targeting the tumor-specific antigen EGFRvIII. Mol. Cancer Ther. *20*, 925–933.
15. Perales-Puchalt, A., Duperret, E.K., Yang, X., Hernandez, P., Wojtak, K., Zhu, X., Jung, S.H., Tello-Ruiz, E., Wise, M.C., Montaner, L.J., et al. (2019). DNA-encoded bispecific T cell engagers and antibodies present long-term antitumor activity. JCI Insight *4*, e126086.

16. Ahmed, N., Salsman, V.S., Kew, Y., Shaffer, D., Powell, S., Zhang, Y.J., Grossman, R.G., Heslop, H.E., and Gottschalk, S. (2010). HER2-specific T cells target primary glioblastoma stem cells and induce regression of autologous experimental tumors. Clin. Cancer Res. *16*, 474–485.

17. Johnson, L.A., Scholler, J., Ohkuri, T., Kosaka, A., Patel, P.R., McGettigan, S.E., Nace, A.K., Dentchev, T., Thekkat, P., Loew, A., et al. (2015). Rational development and characterization of humanized anti-EGFR variant III chimeric antigen receptor T cells for glioblastoma. Sci. Transl Med. *7*, 275ra222.

18. Patel, A., Park, D.H., Davis, C.W., Smith, T.R.F., Leung, A., Tierney, K., Bryan, A., Davidson, E., Yu, X., Racine, T., et al. (2018). In vivo delivery of synthetic human DNA-encoded monoclonal antibodies protect against ebolavirus infection in a mouse model. Cell Rep. *25*, 1982–1993.e4.

19. King, M.A., Covassin, L., Brehm, M.A., Racki, W., Pearson, T., Leif, J., Laning, J., Fodor, W., Foreman, O., Burzenski, L., et al. (2009). Human peripheral blood leucocyte non-obese diabetic-severe combined immunodeficiency interleukin-2 receptor gamma chain gene mouse model of xenogeneic graft-versus-host-like disease and the role of host major histocompatibility complex. Clin. Exp. Immunol. *157*, 104–118.

20. Trimble, C.L., Morrow, M.P., Kraynyak, K.A., Shen, X., Dallas, M., Yan, J., Edwards, L., Parker, R.L., Denny, L., Giffear, M., et al. (2015). Safety, efficacy, and immunogenicity of VGX-3100, a therapeutic synthetic DNA vaccine targeting human papillomavirus 16 and 18 E6 and E7 proteins for cervical intraepithelial neoplasia 2/3: a randomised, double-blind, placebo-controlled phase 2b trial. Lancet *386*, 2078–2088.

21. Hollenberg, R.K., Krieger, D.R., Samuels, R., Kraynyak, K., Sylvester, A., Morrow, M., Boyer, J., Dallas, M., and Bhuyan, P.K. (2020). Safety and immunogenicity of VGX-3100 formulations in a healthy young adult population. Hum. Vaccin. Immunother. *16*, 1404–1412.

22. Betts, M.R., Brenchley, J.M., Price, D.A., De Rosa, S.C., Douek, D.C., Roederer, M., and Koup, R.A. (2003). Sensitive and viable identification of antigen-specific CD8+ T cells by a flow cytometric assay for degranulation. J. Immunol. Methods *281*, 65–78.

23. Han, S., Zhang, C., Li, Q., Dong, J., Liu, Y., Huang, Y., Jiang, T., and Wu, A. (2014). Tumour-infiltrating CD4(+) and CD8(+) lymphocytes as predictors of clinical outcome in glioma. Br. J. Cancer *110*, 2560–2568.

24. Tay, R.E., Richardson, E.K., and Toh, H.C. (2021). Revisiting the role of CD4(+) T cells in cancer immunotherapy-new insights into old paradigms. Cancer Gene Ther. *28*, 5–17.

25. Chen, Z., and Hambardzumyan, D. (2018). Immune microenvironment in glioblastoma subtypes. Front. Immunol. *9*, 1004.

26. Butler, M., Prasad, S., and Srivastava, S.K. (2020). Targeting glioblastoma tumor microenvironment. Adv. Exp. Med. Biol. *1296*, 1–9.

27. Patel, A., Bah, M.A., and Weiner, D.B. (2020). In vivo delivery of nucleic acid-encoded monoclonal antibodies. BioDrugs. *34*, 273–293.

28. Dreier, T., Baeuerle, P.A., Fichtner, I., Grün, M., Schlereth, B., Lorenczewski, G., Kufer, P., Lutterbüse, R., Riethmüller, G., Gjorstrup, P., and Bargou, R.C. (2003). T cell costimulus-independent and very efficacious inhibition of tumor growth in mice bearing subcutaneous or leukemic human B cell lymphoma xenografts by a CD19-/CD3- bispecific single-chain antibody construct. J. Immunol. *170*, 4397–4402.

29. Friedrich, M., Raum, T., Lutterbuese, R., Voelkel, M., Deegen, P., Rau, D., Kischel, R., Hoffmann, P., Brandl, C., Schuhmacher, J., et al. (2012). Regression of human prostate cancer xenografts in mice by AMG 212/BAY2010112, a novel PSMA/CD3-Bispecific BiTE antibody cross-reactive with non-human primate antigens. Mol. Cancer Ther. *11*, 2664–2673.

30. Pillarisetti, K., Powers, G., Luistro, L., Babich, A., Baldwin, E., Li, Y., Zhang, X., Mendonça, M., Majewski, N., Nanjunda, R., et al. (2020). Teclistamab is an active T cell-redirecting bispecific antibody against B-cell maturation antigen for multiple myeloma. Blood Adv. *4*, 4538–4549.

31. Harding, F.A., Stickler, M.M., Razo, J., and DuBridge, R.B. (2010). The immunogenicity of humanized and fully human antibodies: residual immunogenicity resides in the CDR regions. MAbs *2*, 256–265.

32. Davda, J., Declerck, P., Hu-Lieskovan, S., Hickling, T.P., Jacobs, I.A., Chou, J., Salek-Ardakani, S., and Kraynov, E. (2019). Immunogenicity of immunomodulatory, antibody-based, oncology therapeutics. J. Immunother. Cancer *7*, 105.

33. Patel, A., DiGiandomenico, A., Keller, A.E., Smith, T.R.F., Park, D.H., Ramos, S., Schultheis, K., Elliott, S.T.C., Mendoza, J., Broderick, K.E., et al. (2017). An engineered bispecific DNA-encoded IgG antibody protects against Pseudomonas aeruginosa in a pneumonia challenge model. Nat. Commun. *8*, 637.

34. Gantke, T., Weichel, M., Herbrecht, C., Reusch, U., Ellwanger, K., Fucek, I., Eser, M., Müller, T., Griep, R., Molkenthin, V., et al. (2017). Trispecific antibodies for CD16A-directed NK cell engagement and dual-targeting of tumor cells. Protein Eng. Des. Sel. *30*, 673–684.

35. Tapia-Galisteo, A., Sánchez Rodríguez, Í., Aguilar-Sopeña, O., Harwood, S.L., Narbona, J., Ferreras Gutierrez, M., Navarro, R., Martín-García, L., Corbacho, C., Compte, M., et al. (2022). Trispecific T-cell engagers for dual tumor-targeting of colorectal cancer. Oncoimmunology *11*, 2034355.

OMTO, Volume *28*

Supplemental information

Multivalent *in vivo* delivery of DNA-encoded bispecific T cell engagers effectively controls heterogeneous GBM tumors and mitigates immune escape

Daniel H. Park, Kevin Liaw, Pratik Bhojnagarwala, Xizhou Zhu, Jihae Choi, Ali R. Ali, Devivasha Bordoloi, Ebony N. Gary, Ryan P. O'Connell, Abhijeet Kulkarni, Diana Guimet, Trevor Smith, Alfredo Perales-Puchalt, Ami Patel, and David B. Weiner

Figure S1

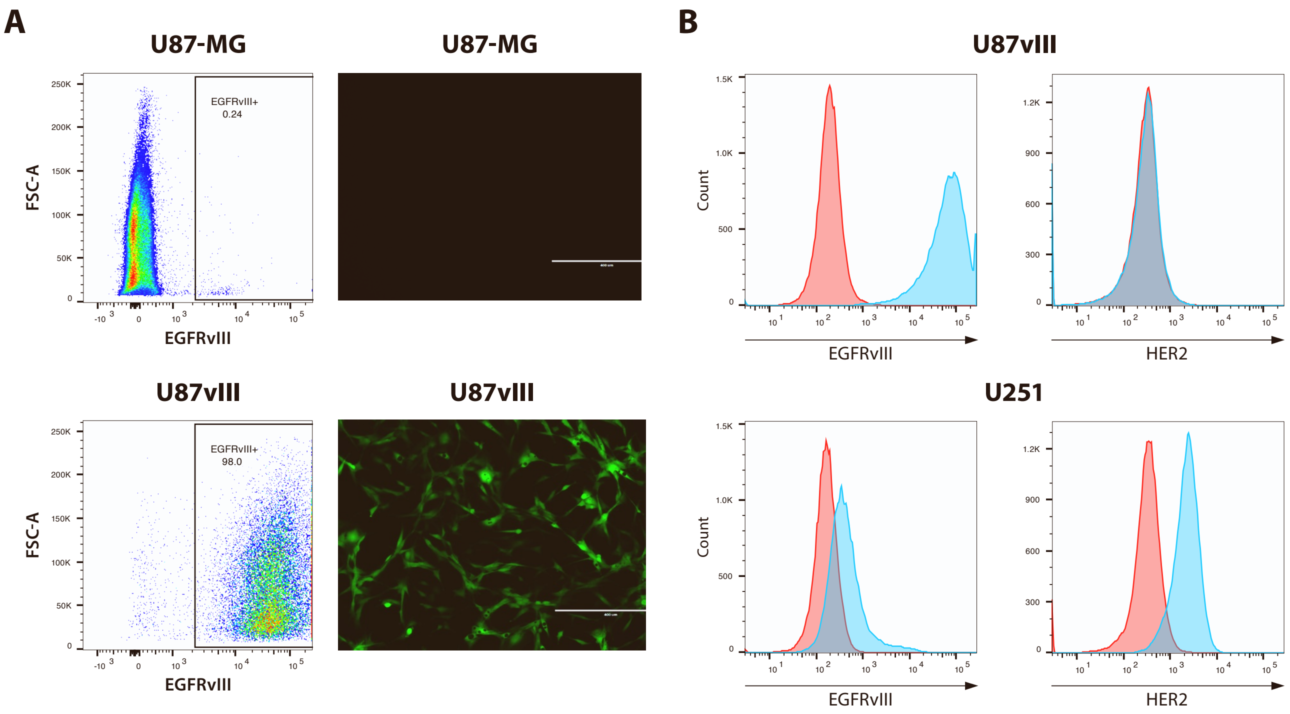

Figure S1. GBM cell lines and antigen expression

(A) Flow cytometry data and fluorescent images showing EGFRvIII expression in wildtype U87 cells and U87vIII cells. (B) Flow cytometry data showing EGFRvIII and HER2 expression in U87vIII cells and U251 cells.

Figure S2

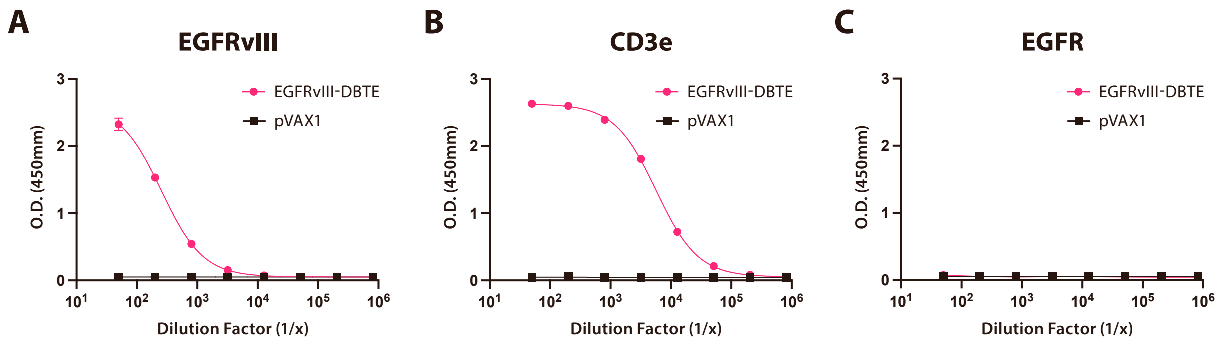

Figure S2. Binding ELISA of EGFRvIII-DBTE

Binding ELISA of EGFRvIII-DBTE (transfection supernatant) against recombinant (A) EGFRvIII, (B) CD3e, and (C) wildtype EGFR.

Video S1

Video S1. Time-lapse video of T cell-mediated cytotoxicity of EGFRvIII-DBTE

Time-lapse video of T cell mediated cytotoxicity against U87vIII cells upon addition of day-14 serum of NSG mouse treated with pVAX1 or EGFRvIII-DBTE. U87vIII is shown in green (GFP). Caspase-3 induction is shown in blue. CD69 activation is shown in red. Hour marks at the top left corner indicate incubation time after addition of T cells and mouse sera.
